# Fatigue and Depression in Sick-Listed Chronic Low Back Pain Patients

**DOI:** 10.1111/pme.12435

**Published:** 2014-04-09

**Authors:** Hildegun Snekkevik, Hege R Eriksen, Tone Tangen, Trudie Chalder, Silje E Reme

**Affiliations:** * FriskvernklinikkenAsker, Norway; † Uni Health, Uni Research ASBergen, Norway; ‡ Department of Psychiatry, Haukeland University HospitalBergen, Norway; § Department of Psychological Medicine, King's College LondonLondon, UK; ¶ Department of Environmental Health, Harvard School of Public HealthBoston, Massachusetts, USA

**Keywords:** Low Back Pain, Fatigue, Depression, Disability

## Abstract

**Objective:**

The relationship between fatigue and pain has been investigated previously, but little is known about the prevalence of substantial fatigue in patients sick-listed for chronic low back pain (CLBP) and about how fatigue is associated with depression, pain, and long-term disability. The aims of the study were to examine the prevalence of substantial fatigue; associations between fatigue, depression, and pain; and whether fatigue predicted long-term disability.

**Methods:**

Five hundred sixty-nine patients participating in a randomized controlled trial and sick-listed 2–10 months for LBP were included in the study. Cross-sectional analyses were conducted to investigate the prevalence and independent associations between fatigue, depression, pain, and disability, while longitudinal analyses were done to investigate the association between fatigue and long-term disability.

**Results:**

The prevalence of substantial fatigue was 69.7%. Women reported significantly more fatigue than men (*t* = −3.6, df = 551; *P* < .001). Those with substantial fatigue had higher pain intensity (*t* = −3.3, df = 534; *P* = 0.01), more depressive symptoms (*t* = −10.9, df = 454; *P* < 0.001), and more disability (*t* = −7.6, df = 539; *P* < 0.001) than those without substantial fatigue. Musculoskeletal pain and depression were independently associated with substantial fatigue. In the longitudinal analyses, fatigue predicted long-term disability at 3, 6, and 12 months' follow-up. After pain and depression were controlled for, fatigue remained a significant predictor of disability at 6 months' follow-up.

**Conclusions:**

The vast majority of the sick-listed CLBP patients reported substantial fatigue. Those with substantial fatigue had more pain and depressive symptoms and a significant risk of reporting more disability at 3, 6, and 12 months. Substantial fatigue is disabling in itself but also involves a risk of developing chronic fatigue syndrome and long-term disability.

## Introduction

Chronic low back pain (CLBP) represents a major public health problem in Western societies. It is the leading cause of long-term sick leave in Norway and is often associated with substantial disability [1],[2] and a high degree of comorbidity. The comorbidity involves both mental disorders [3] and other health complaints [4], including tiredness and fatigue [5].

Fatigue is a subjective health complaint that entails emotional, cognitive, and behavioral components [6]. The current study will be covering the mental and physical aspects of fatigue [7], as described and applied in previous studies [8],[9]. More specifically, these involve cognitive difficulties, tiredness and sleepiness, reduced strength and endurance, and loss of interest and motivation [10].

Fatigue is a common complaint in the general population [8],[9]. It often accompanies physical diseases 11–14 and psychiatric disorders [15] and is the most prominent and severe symptom in chronic fatigue syndrome (CFS) [16]. Patients with various pain syndromes also report fatigue. A higher occurrence of fatigue was found in patients with complex regional pain syndrome compared with a control group [17], and patients with fibromyalgia were more fatigued than the general population [18]. Two previous studies have shown that CLBP patients are more fatigued than healthy controls [19],[20], but less is known about the prevalence of *substantial* fatigue in CLBP compared with the general population and about how fatigue may influence the prognosis of CLBP.

Depression is a factor known to influence the prognosis of CLBP [21]. In fact, depression is both a risk factor for [22] and a consequence of chronic pain [23], and patients with concurrent pain and depression have more intense and continuous pain, more functional limitations, and slower recovery [23],[24]. The presence of depressive symptoms is also strongly associated with chronic fatigue [25], and depression could thus be a contributing etiological factor of the reported fatigue in chronic pain patients.

A related problem, clinical insomnia, is also highly prevalent among patients with chronic back pain [26]. A study from a large, heterogeneous sample of patients who were seeking care for their LBP revealed that 59% of the patients reported poor sleep [27]. Sleep is thus increasingly recognized as an important parameter in determining quality of life in chronic pain patients and could be an underlying factor of the fatigue in CLBP.

The pain itself could also be a contributing cause of the fatigue, with fatigue and pain both contributing to a synergistic reduction of functional capacity in CLBP. Co-occurrence of fatigue and CLBP may thus contribute to a greater loss of function [3] as well as an increased risk for sick leave [28].

The primary aim of this study was to investigate the prevalence of substantial fatigue, defined as a score of ≥4 on the Chalder Fatigue Scale [7], in sick-listed CLBP patients. Secondary aims involved assessing associations between fatigue, depression, pain, subjective health complaints, and disability and exploring how fatigue influenced the prognosis at 3, 6, and 12 months' follow-up. We hypothesized that substantial fatigue would be associated with more widespread and intense musculoskeletal health complaints and depression and that pain, depression, and fatigue would predict disability after 3, 6, and 12 months.

## Methods

A total of 569 patients who were sick-listed with nonspecific LBP for between 2 and 10 months were included in the study as part of a multicenter randomized controlled trial. Details are described in the trial protocol [29]. The work was conducted at Uni Health, Uni Research AS, Bergen, Norway. Patients on sick leave for nonspecific LBP (International Classification of Primary Care [WHO, 2003]: L03, L04, L84, and L86) received information from the Norwegian Labour and Welfare Administration about the possibility of participating in a multicenter randomized controlled trial of different treatments for CLBP. Those who responded to the invitation (N = 2,200) were screened by telephone and excluded if they did not fulfill the following inclusion criteria: currently being on sick leave due to LBP and having been so for between 2 and 10 months, being between 18 and 60 years of age, and being fluent in the Norwegian language. A total of 1,563 patients were excluded at this stage. The remaining 637 patients were referred to the participating clinics for inclusion, and 63 were excluded due to the following exclusion criteria: pregnancy (N = 1), known osteoporotic fracture or being on antiosteoporotic medication (N = 5), ongoing treatment for cancer (N = 2), recent back trauma (N = 11), serious psychiatric disorder (mainly due to ongoing psychosis, high suicide risk, and/or serious depression; N = 8), cardiovascular disease (N = 1), and nonfluency in the Norwegian language (N = 6). A standardized psychiatric interview was used to screen for psychiatric disorders. One patient withdrew his consent and demanded that all data be deleted, and another 4 patients were excluded after randomization, leaving 569 patients included in the analyses.

The patients answered a number of different questionnaires at baseline and at 3, 6, and 12 months' follow-up concerning pain, fatigue, anxiety, depression, subjective health complaints, and functional limitations. In addition, demographic variables such as gender, age, civil status, and education were recorded. The response rates for the follow-up assessments were 55% at 3 months, 49% at 6 months, and 68% at 12 months.

### Questionnaires

#### Pain

Mean back pain severity during the last 14 days was registered using a visual analog rating scale (VAS scale) divided into 11 equal parts with scores from 0 to 10, with 0 labeled “no pain” and ten “worst possible pain.” The patients were also asked to mark on a body chart where they had experienced pain during the last week and to rate the intensity of neck pain and leg pain on VAS scales.

#### Depression

The Hospital Anxiety and Depression Scale (HADS) has been shown to be a useful device for assessing presence of clinically significant degrees of anxiety and depression [30]. The HADS is a 14-item scale and is scored on a four-point Likert scale. It was originally developed for use in patients with physical illness, with excluded items concerning somatic symptoms in anxiety and depression. The scale hence avoids overlap with somatic symptoms of physical illness. Fourteen items measuring anxiety and depression over the last week (seven each) provide a total score of between 0 and 21 for each subscale. Higher scores on these subscales represent increased probability of an anxiety or depressive disorder being present. We only used the depression subscale in the current study, with scores of 8 and above being used as an indication of possible depression [31].

#### Fatigue

The Chalder Fatigue Scale [7] is a self-rating scale developed to measure the severity of fatigue. The intended purpose of the scale is to detect fatigue cases and to assess symptom severity in both hospital and community populations. It is an 11-item scale and has been found to be both reliable and valid [7],[32]. The patients are asked to rate how tired they have or have not been over the last month, with the following response alternatives: “better than usual,” “no more than usual,” “worse than usual,” and “much worse than usual”; the higher the score, the more fatigued the patient. If participants had been feeling tired for a long time, they were asked to compare their tiredness with how they felt when last well. The bimodal scoring system allows for fatigue cases to be identified. In contrast to the Likert scoring system (0, 1, 2, 3), where the maximum score possible is 33, the bimodal scoring system (0, 0, 1, 1) has a maximum score of 11, where the response options “less than usual” and “no more than usual” are given scores of 0 and “worse than usual” and “much worse than usual” are given scores of 1. “Substantial fatigue” was defined by total dichotomized scores of 4 or higher. This cutoff was originally suggested by a previous validation study of the Chalder Fatigue Scale [7] and had since been applied in two population studies, thus providing norms that could be used for comparison [8],[9].

#### Subjective Health Complaints

The Subjective Health Complaints (SHC) inventory [33] is a 29-item questionnaire designed to measure prevalence, degree, and duration of common subjective health complaints. The patients are asked if and, if yes, to what extent they have been affected by any of 29 complaints during the last month, using the following options: 0 (not at all), 1 (a little), 2 (some), or 3 (seriously). The 29 items may be categorized into five subscales: musculoskeletal complaints (headache, neck pain, shoulder pain, pain in upper back, low back pain, pain in arms, leg pain, migraine), pseudoneurological conditions (extra heartbeats, hot flashes, sleep problems, tiredness, dizziness, anxiety, sadness/depression), gastrointestinal complaints (heartburn, stomach discomfort, ulcer/nonulcer dyspepsia, stomach pain, gas discomfort, diarrhea, constipation), allergy (asthma, breathing difficulties, eczema, allergy, chest pain), and flu (cold/flu, coughing). In the current study, severity of complaints was also computed for each subscale.

#### Disability

The Oswestry Disability Index [34] was used to assess functional limitation or disability at baseline and at 3-, 6-, and 12-month follow-up. It consists of 10 items concerning the effect of back pain on different activities of daily life (personal care, lifting, walking, sitting, standing, sleeping, sexual life, social life, and travelling). The patients are asked about their current functional status. Each item is scored from 0 to 5, with higher values representing more disability. In the current study we used a median split and defined those in the upper half (with a score of >28) as substantially disabled.

### Statistical Analyses

All analyses were performed with SPSS version 18. Descriptive statistics, involving frequency tables, cross-tabs, and independent-sample *t*-tests, was used to assess the prevalence of substantial fatigue and to compare differences between groups, i.e., those with and without substantial fatigue. Multiple logistic regression was used to investigate associations between the variables and possible confounders, both cross-sectionally and longitudinally. Additional correlation analyses were conducted between the independent variables to test for potential multicollinearity.

### Ethical Considerations

The Regional Ethical Committee and the Norwegian Social Science Data Services National Register of Data approved the study. All principles in the Helsinki declaration were followed. Informed consent was signed by each participant with emphasis on the right to withdraw from the study at any time without any explanation.

## Results

The study population was 50.3% women, and the mean age was 44 years. Patients reported an average of 11 years' duration for their back pain (Table 1), with 99.6% reporting pain lasting for more than 3 months. Three hundred seventy-eight (69.7%) reported substantial fatigue, with a score of 4 or more on the Chalder Fatigue Scale. Women reported significantly higher scores on fatigue compared with men (*t* = −3.6, df = 551; *P* < .001). One hundred one patients (18%) were possibly depressed, with a score of 8 or more on the HADS depression subscale. No gender differences were seen for depression (χ^2^ = 0.06, *P* = 0.44).

**Table 1:** Baseline and clinical characteristics (N = 569)

Continuous variables	Mean	SD	Median
Age	44.3 years	9.7	44
Duration of back pain	10.8 years	10.5	7.5
Back pain intensity (0–10)	6.5	1.9	7.0
Neck pain intensity (0–10)	3.8	2.8	4.0
Leg pain intensity (0–10)	3.9	2.7	4.0
Pain during activity (0–10)	5.9	2.2	6.0
Pain while resting (0–10)	4.0	2.3	4.0
Subjective health complaints (number of complaints)	10.0	4.9	10.0

Categorical variables	N	%
Gender		
Men	283	49.7
Women	286	50.3
Civil status		
Married/cohabiting	396	71.7
Single/widow/divorced	156	28.3
Education		
Primary school, 1–12 years	386	70.2
University/college	164	29.8
HADS		
Depression (score ≥8)	101	18.3
Anxiety (score ≥8)	125	22.6
Subjective health complaints		
Musculoskeletal complaints	528	99.2
Pseudoneurological complaints	477	89.7
Gastrointestinal complaints	360	67.5
Allergy	243	45.6
Flu	228	42.9

HADS = Hospital Anxiety and Depression Scale.

Patients with substantial fatigue had higher pain intensity (*t* = −3.3, df = 534; *P* = 0.01), more depressive symptoms (*t* = −10.9, df = 454; *P* < 0.001), and more disability (*t* = −7.6, df = 539; *P* < 0.001) compared with those without substantial fatigue. Seventeen percent of the CLBP patients were both substantially fatigued (≥4 on fatigue scale) and scored above the cutoff for depressive symptoms (≥8 on HADS).

In the univariate models, female gender, back pain, leg pain, anxiety, depression, and the SHC subscales for gastrointestinal problems, flu, and musculoskeletal pain were significantly associated with substantial fatigue. Higher scores were associated with substantial fatigue (Table 2).

**Table 2:** Univariate and multivariate associations between substantial fatigue and other clinical and demographic variables

	Univariate associations		Multivariate model	
OR (95% CI)	*P* value	OR (95% CI)	*P* value
Age	1.00 (0.98–1.02)	0.80	0.98 (0.95–1.00)	0.06
Marital status	0.79 (0.52–1.20)	0.27	0.87 (0.51–1.48)	0.60
Education	1.25 (0.83–1.88)	0.29	1.07 (0.63–1.80)	0.80
Gender	2.02 (1.39–2.94)	<0.001	1.62 (1.00–2.63)	0.05
Back pain intensity	1.18 (1.07–1.30)	0.001	0.97 (0.85–1.11)	0.68
Leg pain intensity	1.15 (1.07–1.24)	<0.001	1.10 (1.01–1.21)	0.04
Anxiety (HADS anxiety scale)	1.30 (1.21–1.40)	<0.001	1.09 (0.98–1.21)	0.10
Depression (HADS depression scale)	1.41 (1.30–1.53)	<0.001	1.28 (1.15–1.43)	<0.001
Gastrointestinal problems (SHC)	1.16 (1.06–1.26)	0.001	0.96 (0.85–1.08)	0.46
Flu (SHC)	1.31 (1.10–1.56)	0.03	1.21 (1.00–1.48)	0.053
Musculoskeletal pain (SHC)	1.29 (1.21–1.37)	<0.001	1.22 (1.13–1.31)	<0.001

Outcome variable is substantial fatigue (≥4 on the Chalder Fatigue Scale).

OR = odds ratio; CI = confidence interval; HADS = Hospital Anxiety and Depression Scale; SHC = Subjective Health Complaints inventory.

In the multivariate model, gender, age, civil status, and length of education were included as control variables. The pseudoneurological conditions subscale in the SHC inventory was highly correlated with depression (*r* = 0.51, *P* < .001) and was thus excluded from the final model in order to avoid multicollinearity. In the final model, higher scores on the depression scale, musculoskeletal pain, and leg pain were significantly and independently associated with substantial fatigue (see Table 2).

The same model was run with disability as outcome in order to test another hypothesis, namely that stronger pain, fatigue, and depressive symptoms were associated with substantial disability (score >28 on the Oswestry Disability Index). Higher scores for fatigue, depressive symptoms, and musculoskeletal pain (including back and leg pain) were significantly and independently associated with substantial disability (see Table 3).

**Table 3:** Univariate and multivariate associations between functional disability and other clinical and demographic variables

	Univariate associations		Multivariate model	
OR (95% CI)	*P* value	OR (95% CI)	*P* value
Age	1.00 (0.98–1.01)	0.68	0.99 (0.97–1.01)	0.39
Marital status	0.74 (0.51–1.07)	0.11	0.78 (0.49–1.24)	0.29
Education	0.72 (0.50–1.05)	0.09	0.84 (0.53–1.33)	0.46
Gender	1.19 (0.85–1.66)	0.32	1.06 (0.69–1.63)	0.80
Fatigue	3.59 (2.38–5.39)	<0.001	2.43 (1.43–4.14)	0.001
Back pain intensity	1.52 (1.36–1.70)	<0.001	1.38 (1.21–1.58)	<0.001
Leg pain intensity	1.23 (1.15–1.32)	<0.001	1.13 (1.05–1.23)	0.002
Anxiety	1.11 (1.06–1.17)	<0.001	0.95 (0.88–1.03)	0.24
Depression	1.18 (1.11–1.24)	<0.001	1.13 (1.03–1.23)	0.007
Gastrointestinal problems (SHC)	1.15 (1.07–1.23)	<0.001	1.04 (0.95–1.14)	0.38
Flu (SHC)	1.16 (1.01–1.33)	0.04	1.13 (0.96–1.33)	0.15
Musculoskeletal pain (SHC)	1.16 (1.11–1.22)	<0.001	1.06 (1.00–1.13)	0.058

Outcome variable is disability (>28 on the Oswestry Disability Index).

OR = odds ratio; CI = confidence interval; SHC = Subjective Health Complaints inventory.

Finally, the longitudinal analyses showed that fatigue predicted disability at 3, 6, and 12 months' follow-up when demographic variables and baseline level of disability were controlled for. When pain and depression were added as potential covariates, fatigue remained a significant predictor of disability at 6 months (Table 4).

**Table 4:** Fatigue as predictor of functional disability at 3, 6, and 12 months with different levels of adjustments for covariates

	Model†	3 months' follow-up		6 months' follow-up		12 months' follow-up	
	OR (95% CI)	*P* value	OR (95% CI)	*P* value	OR (95% CI)	*P* value
Fatigue (0–11)	A	1.29 (1.18–1.41)	0.000	1.30 (1.17–1.45)	0.000	1.20 (1.11–1.30)	0.000
		B	1.19 (1.07–1.33)	0.002	1.25 (1.09–1.43)	0.001	1.11 (1.01–1.23)	0.036
		C	1.18 (1.06–1.31)	0.003	1.21 (1.07–1.37)	0.003	1.09 (0.99–1.21)	0.093
		D	1.11 (0.98–1.26)	0.099	1.19 (1.02–1.39)	0.031	1.03 (0.92–1.16)	0.567
Fatigue (dichotomous)	A	4.22 (2.25–7.93)	0.000	5.73 (2.50–13.2)	0.000	2.63 (1.45–4.77)	0.001
		B	2.30 (1.10–4.82)	0.027	4.04 (1.51–10.8)	0.005	1.32 (0.65–2.66)	0.443
		C	2.43 (1.20–4.91)	0.013	3.83 (1.58–9.28)	0.003	1.45 (0.74–2.84)	0.279
		D	1.61 (0.73–3.56)	0.235	3.07 (1.13–8.38)	0.028	0.89 (0.42–1.91)	0.764

Outcome variable is disability (>28 on the Oswestry Disability Index).

† A = unadjusted; B = adjusted for disability, age, gender and education; C = adjusted for pain and depression; D = model C + model B.

## Discussion

A total of 70% of the 569 patients with CLBP reported substantial fatigue (dichotomized scores 4 or higher) in the current study. More women than men were fatigued. Those who were substantially fatigued had higher pain intensity, more depressive symptoms, and more functional disability than those without substantial fatigue. In the multiple regression models, musculoskeletal pain and depression were independently associated with substantial fatigue, while pain intensity, depression, and fatigue were independently associated with substantial disability. This implies that the fatigue is fully explained neither by depression nor by pain intensity. The longitudinal analyses further confirmed that the severity of fatigue was a significant predictor of disability at 3, 6 and 12 months' follow-up. This association remained significant at 6 months' follow-up when pain intensity and depression were controlled for.

The prevalence of substantial fatigue reported here is far higher than in the general Norwegian population, where 22% reported substantial fatigue [9]. It is also higher than what was reported in the general British population, where 37% reported substantial fatigue [8]. Our finding of more fatigue in women than in men is, however, in accordance with previous findings [8]. Our results are further in agreement with two previous studies on CLBP and fatigue, where the CLBP patients reported significantly more fatigue than the healthy controls [19],[20]. Compared with other patient populations, more CLBP patients than patients with rheumatoid arthritis (42%) and osteoarthritis (41%) report substantial fatigue, but the number who do so is comparable with that of patients with fibromyalgia (76%) [35]. The prevalence of clinically relevant fatigue in patients with different cancer diagnoses has been found to vary from 19% (testicular cancer) to 38% (breast cancer) [13].

Pain is one common aspect of rheumatoid arthritis, osteoarthritis, fibromyalgia, and LBP, and one might hypothesize that pain is a contributing cause of the fatigue. Three studies of diabetic patients with peripheral neuropathic pain supported this hypothesis; after treatment with duloxetine, the patients reported less pain and improvements in perceived fatigue 36–38. A path analysis of the results further suggested that fatigue improvement was mediated by reductions in pain, night pain, and sleep interference [39]. Thus, although not able to confirm a causal link between reduced pain and improved fatigue, findings indicate that reduced fatigue may be secondary to improvements in pain [40] and that the relationship may be etiological [41]. The current study provides some support for this notion in that the predictive value of fatigue was reduced when pain and depression were added as covariates in the longitudinal analyses.

Eighteen percent of the CLBP patients scored high on depressive symptoms, with a score of 8 or more on the HADS depression subscale. This was not unexpected given the close relationship between pain and depression seen in previous literature 42–45. A score of 8 or more on the HADS depression subscale has previously been found to be a strong predictor of both work disability and mortality [46],[47]. We found that depressive symptoms were strongly correlated with fatigue, and a subgroup of 17% reported both substantial fatigue and depressive symptoms. Comorbid fatigue and depression might reduce the patients' functional capacity, and this synergistic effect of what are usually referred to as “symptom clusters” has been thoroughly demonstrated [48],[49].

Substantial fatigue is also a predictor of chronic fatigue and CFS [50],[51]. Although a lack of CFS screening in the study makes us unable to rule out already existent CFS, the reported substantial fatigue could still represent a risk factor, indicating a need for fatigue screening at an early stage of CLBP. Fatigue was a strong predictor of long-term disability, which implies that fatigue and its consequences may need to be considered when CLBP treatment programs are planned. Indeed, multimodal treatment programs for patients with both CLBP and fatigue have indicated good results [52]. Further, reducing pain may in itself contribute to less fatigue, particularly night pain and pain interfering with sleep [39]. Also, addressing the fatigue directly in rehabilitation programs may give additional effects, as cognitive–behavioral therapy and graded exercise therapy have produced significant improvements in fatigue for patients with CFS [53],[54].

The study has a few limitations that will now be considered. First, the participants were not formally screened for CFS, fibromyalgia, or sleep disorders. These conditions can therefore not be ruled out as underlying causes of the fatigue, thereby calling into question the representativeness of the CLBP population. However, the fact that all study participants were on sick leave due to LBP implies that they were representative of this population by definition. Although an underlying comorbid diagnosis of, e.g., CFS cannot be ruled out, this was not their primary reason for being unable to work, and it was not their primary reason for seeking treatment. A systematic screening for CFS in this population would, however, be interesting and should be pursued in future studies. Second, the use of different fatigue scales might be a problem when comparing fatigue across studies. Still, the most important comparison for the current study were the studies from the general population, which used both the same scale and cutoff as in the current study [8],[9]. Last, loss to follow-up is also a limitation that needs to be considered. The longitudinal analyses, especially at 6 months' follow-up, suffer from a high rate of missing data, and these results should thus be interpreted with some care.

In conclusion, we found that the vast majority of sick-listed CLBP patients reported substantial fatigue, and more so for women than for men. Musculoskeletal pain and depression were associated with substantial fatigue, while intense pain, depression, and fatigue were associated with substantial disability. The patients with substantial fatigue had higher pain intensity and more disability and were more depressed than those without, and severity of fatigue was a significant predictor of long-term disability.
